# Safety and Dignity in Older Adult Mental Healthcare: Embedding Gerontological Innovation in Practice

**DOI:** 10.1093/geroni/igaf122.3842

**Published:** 2025-12-31

**Authors:** Kristine Lee, Katarina Friberg-Felsted

**Affiliations:** University of Utah College of Nursing, Cedar Hills, Utah, United States; University of Utah, Salt Lake City, Utah, United States

## Abstract

The growing demographic of older adults calls for urgent considerations for age-friendly outpatient and community mental health services on their behalf. Rates of depression and anxiety are high in later life, yet few clinicians receive gerontological training. This lack of expertise undermines quality and safety. Although cognitive behavioral therapy (CBT) and reminiscence therapy are effective in older populations, they remain underutilized. Workforce shortages intensify these gaps: fewer than 4% of psychologists and 2% of social workers pursue advanced training in gerontology. The expansion of ketamine for treatment-resistant depression and anxiety illustrates the risks of innovation outpacing evidence. While FDA-approved for adults with depression under strict REMS monitoring, the scientific literature regarding ketamine use in older adults is minimal. Published data consist primarily of case reports and one small pilot study in mild cognitive impairment; there are no randomized controlled trials in Alzheimer’s disease, dementia syndromes, or residential care populations. Known adverse effects—dissociation, hypertension, sedation, increased fall risk, and delirium are particularly concerning. In the absence of gerontological expertise, off-label use in outpatient or assisted-living settings risks compromising patient safety. This project proposes an evidence-based framework for outpatient mental health care in aging populations. Drawing on Erikson’s psychosocial theory, Butler’s life review model, and Pearlin’s stress process model, the Gerontological Mental Health Framework integrates CBT, reminiscence therapy, psychoeducation, and caregiver support groups. Interdisciplinary staffing, including geropsychiatrists, gerontologists, social workers, and caregiver peer facilitators, ensures developmentally informed, team-based care. This scalable framework addresses geropsychology, offering strategies to strengthen outpatient mental health services.

